# A Method for Measuring Shaft Diameter Based on Light Stripe Image Enhancement

**DOI:** 10.3390/s24010303

**Published:** 2024-01-04

**Authors:** Chunfeng Li, Xiping Xu, Siyuan Liu, Zhen Ren

**Affiliations:** 1 College of Optoelectronic Engineering, Changchun University of Science and Technology, Changchun 130022, China; 2 School of Electronic Information Engineering, Changchun University, Changchun 130022, China; 3 School of Mechanical and Aerospace Engineering, Jilin University, Changchun 130022, China; siyliu@jlu.edu.cn; 4 College of Mechanical and Vehicle Engineering, Changchun University, Changchun 130022, China

**Keywords:** shaft diameter measurement, line structured light, image enhancement, machine vision

## Abstract

When the workpiece surface exhibits strong reflectivity, it becomes challenging to obtain accurate key measurements using non-contact, visual measurement techniques due to poor image quality. In this paper, we propose a high-precision measurement method shaft diameter based on an enhanced quality stripe image. By capturing two stripe images with different exposure times, we leverage their different characteristics. The results extracted from the low-exposure image are used to perform grayscale correction on the high-exposure image, improving the distribution of stripe grayscale and resulting in more accurate extraction results for the center points. The incorporation of different measurement positions and angles further enhanced measurement precision and robustness. Additionally, ellipse fitting is employed to derive shaft diameter. This method was applied to the profiles of different cross-sections and angles within the same shaft segment. To reduce the shape error of the shaft measurement, the average of these measurements was taken as the estimate of the average diameter for the shaft segment. In the experiments, the average shaft diameters determined by averaging elliptical estimations were compared with shaft diameters obtained using a coordinate measuring machine (CMM) the maximum error and the minimum error were respectively 18 μm and 7 μm; the average error was 11 μm; and the root mean squared error of the multiple measurement results was 10.98 μm. The measurement accuracy achieved is six times higher than that obtained from the unprocessed stripe images.

## 1. Introduction

As a rotating component, shafts are one of the most common types of parts in mechanical equipment. Generally, shafts consist of several cylindrical sections with different diameters, and their length is greater than their diameter. They play a crucial role in supporting other components, bearing loads, and transmitting torque [1,2]. In order to achieve proper coordination with other parts and optimize the performance of shafts, it is essential to strictly control the machining accuracy of their design dimensions, especially of critical dimensions such as shaft diameter. High-precision measurement is a prerequisite for high-precision machining. With the introduction and advancement of intelligent manufacturing, intelligent and efficient manufacturing places higher demands on measurement techniques [3,4,5].

In traditional measurement techniques, the dimensions and tolerances of parts are often obtained through contact measurement methods, including coordinate measuring instruments, vernier calipers, and other specialized gauges. Meeting the measurement requirements of parts under non-contact measurement conditions using these methods is challenging [6,7]. Structured light vision measurement technology, which operates based on the principle of laser triangulation, offers a non-contact optical measurement solution. This technology enables fast and efficient measurement of relevant dimensions without touching the workpiece surface [8,9,10,11]. Projection of a structured light pattern onto the surface of the workpiece and capturing the structured light image using a calibrated industrial camera makes it possible to rapidly calculate the actual three-dimensional information of the surface using the camera imaging model [12,13,14,15]. This information can then be used to obtain the desired dimensions of the workpiece. F. Hao et al. [16] derived a diameter-measurement method based on the imaging principle of small holes, similar triangles, and tangent properties using two images with different object distances. Under carefully controlled experimental conditions, this method achieved high measurement accuracy. However, random errors in a single measurement are still a significant concern. Li et al. [17] proposed a precise measurement method for large shaft diameters using a dual-telecentric camera-based measurement system. They developed a calibration method for two telecentric cameras based on Zhang’s calibration method [18] and established a common world coordinate system for the two cameras using the dual-camera imaging model. The diameter-measurement formula was derived from this calibration process. Nogueira et al. [19] presented a comprehensive method for the automated evaluation of planar dimensions of mechanical parts. This method significantly advanced cost-effectiveness, accuracy, and repeatability. They proposed a functional prototype that combined improved subpixel edge-detection methods with planar measurement while maintaining low measurement costs to ensure precise image-based measurements. Liu et al. [20] proposed a method for measuring shaft diameter using structured light vision. After the structured light-based measurement model is calibrated, a virtual plane perpendicular to the axis of the measured shaft is established. The light stripe images on the shaft are projected onto the virtual plane. With the geometric constraints of the light stripes on the virtual plane, the center of the measured shaft is determined by fitting the projected images. The diameter of the shaft is then measured using the determined center and the projected images, resulting in high measurement accuracy and reliability.

In addition to calibration of the measurement system, the key challenge in structured-light vision-measurement technology lies in extracting the center points of the projected light stripes from the acquired images accurately and with high precision [21,22]. The two-dimensional coordinates of the light-stripe center points can be mapped to the accurate three-dimensional coordinates on the surface of the object using the camera-imaging model. Therefore, the accuracy with which the center point of the light stripe is extracted directly affects the final measurement accuracy. Moreover, due to the pinhole-imaging mechanism of the camera, errors in extracting the light-stripe center points from the images will be linearly magnified, with the magnification factor proportional to the object distance during image acquisition. Traditional algorithms for light-stripe center-point extraction include linear interpolation, Gaussian approximation, extremum detection, the centroid method, and the Steger algorithm. Among these methods, the Steger algorithm provides more accurate subpixel coordinates of the light-stripe center points but requires more computational time. However, almost all light-stripe center-point extraction algorithms require the light stripes in the images to be of uniform quality [23,24,25], with good contrast with the background and a gray-level distribution as close as possible to a Gaussian or Gaussian plateau distribution to ensure satisfactory extraction results.

When using structured light to measure diffuse reflection targets with low surface reflectivity, it is relatively easy to meet the requirements mentioned above for the quality of the light stripe images [26,27,28,29]. However, when measuring highly reflective objects, the situation becomes less favorable. The secondary reflections on the surface of highly reflective objects can alter the gray-level distribution of the light stripes in the images, affecting the accuracy of center-point extraction. Additionally, specular reflections can cause overexposure or underexposure of the light stripe images, which further impacts the center-point extraction and leads to inaccurate measurement results.

Due to the characteristics of highly reflective materials, highly reflective and high-contrast areas often appear overexposed on images of highly reflective objects. This phenomenon leads to a decrease in image quality, loss of texture details, and challenges in accurately measuring highly reflective objects using structured-light visual-measurement techniques. Yau [30] proposed a “high-dynamic-range scanning” technique using multiple exposures. A series of images captured at different exposure times is merged into a set of HDR images, and the phase-shifted image selects the brightest non-saturated intensity at each pixel. As the impact of ambient lighting on the phase-shift method is minimal, this approach allows stable measurement of local variations in surface reflectance. However, due to the subjective selection of exposure times, there is a lack of quantitative calculations by which to determine the appropriate exposure time. Additionally, synthesizing HDR images typically requires multiple long-exposure shots, resulting in low measurement efficiency. Chen et al. [31] developed an adaptive stripe image by using a polynomial fitting algorithm to calculate the optimal projected light intensity based on the reflection characteristics of the measured surface. This approach generates an adaptive stripe image that avoids image saturation and achieves a high signal-to-noise ratio (SNR), enabling accurate measurement of the three-dimensional shape of highly reflective surfaces. Because this technique involves capturing a minimal number of images to generate adaptive stripe images, the time-consuming task of capturing a large number of images is avoided. Lin et al. [32] developed an adaptive digital stripe-projection technique for HDR three-dimensional shape measurement. This technique adjusts the intensity of the projected stripe pattern on a pixel-by-pixel basis to handle highly reflective surfaces and improve measurement accuracy. It adapts the intensity of the projection stripe pattern by considering the camera response function, surface reflectance, and illumination from both the environment and surface reflection. Because this approach establishes a novel mathematical model by which to calculate the intensities of environmental light and surface reflections, the optimal intensity for each pixel in the projected stripe pattern can be accurately determined. This method outperforms previously proposed approaches that use different illuminations to synthesize HDR images.

Although there are effective methods in the field of 3D reconstruction for reconstructing highly reflective surfaces, most of these methods are applicable only to stripe projection techniques, and there are few methods that can address the issue of high reflectivity in line-structured light monocular visual-measurement techniques. The traditional approach involves applying a developer to the surface of the measured object to alter its surface reflectance in favor of diffuse reflection, but this method is not only inefficient, but also introduces additional measurement errors. Another method for measuring objects with highly reflective surfaces is to eliminate the influence of highlights by changing the angle between the transmission axes of polarizing filters. Feng et al. [33] proposed three solutions for HDR measurement of three-dimensional shapes based on digital stripe projection to address the measurement challenges posed by highly reflective surfaces. One of the techniques combines the multiple-exposure method with the polarizing-filter method: two orthogonal polarizing filters are placed in front of the camera and projector to measure surfaces with low reflectivity. However, the use of polarizing filters to reduce intensity in the polarized method may result in lower SNR in the captured stripe images. Moreover, this approach increases the complexity of the measurement system’s hardware, as fine adjustments of the angles between the polarizing filters are required to accommodate different working conditions. Such adjustments rely heavily on human expertise and manual techniques. Kowarschik et al. [34] compensated for the influence of reflections from highly reflective surfaces and shadow areas by using 15 optical-projection directions. The object can also be rotated to generate additional views. The camera captures different color blocks on the surface in local coordinates, which are then synthesized into global coordinates. However, the use of numerous system parameters affects the calibration speed. Furthermore, hardware, system settings, image templates, and synthesis processing introduce additional complexities.

To overcome the impact of surface reflections and achieve precise measurement of critical dimensions of reflective parts, a method that utilizes enhanced light stripe images to suppress reflections and improve the quality of the light stripes is proposed. Two light stripe images with different exposure times are captured. The choice of exposure times is based on the characteristics of light stripe images under different exposure conditions. The results obtained from the light stripe image with the lower exposure time are used to perform grayscale correction on the light stripe image with the higher exposure time, thereby improving the distribution of the light stripe grayscale. The center points are then re-extracted to improve the results. The improved center-point coordinates are then mapped to actual three-dimensional data points using the calibrated measurement-system model. Finally, in order to reduce the impact of shape error on the measurement of shaft diameter, an estimate of diameter derived from multiple cross-sections of a shaft segment and multiple contour segments from the same section is obtained through elliptical fitting. The average diameter of the shaft, as determined by averaging elliptical estimations, is taken as the diameter of the shaft segment.

## 2. Methods

### 2.1. Visual System Calibration

#### 2.1.1. The Camera-Based Imaging System

**Figure 1:**
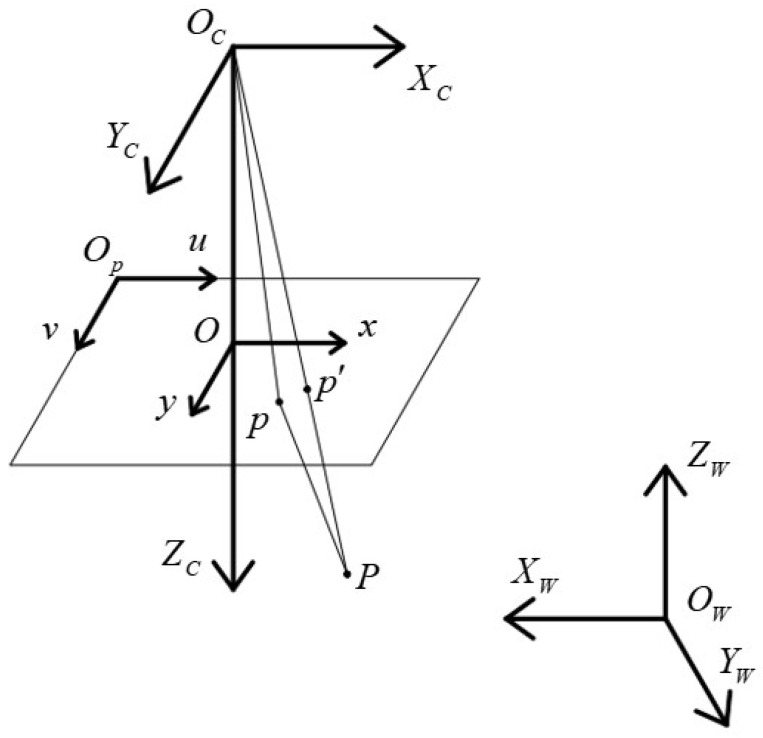
Schematic diagram of the camera-based imaging coordinate relationship.

To fully describe the imaging of a point in space, at least four coordinate systems are required: the world coordinate system $\left(O_{W}X_{W}Y_{W}Z_{W}\right)$, the camera coordinate system $\left(O_{C}X_{C}Y_{C}Z_{C}\right)$, the image coordinate system $\left(Oxy\right)$, and the pixel coordinate system $\left(O_{p}uv\right)$. The relationships between these coordinate systems are shown in Figure 1. The camera’s imaging model can be categorized into linear and nonlinear models based on the presence of distortion. In the linear model, there is a linear projection between the spatial point and the image point. In the nonlinear model, spatial points undergo deviations during the projection process, which are known as distortion.

In the visual system for measuring shaft diameter, the pixel values in the shaft-diameter image do not have a physical meaning, making it difficult to directly obtain dimensional information for the shaft parts. Therefore, the purpose of establishing a camera-based imaging system is to project the information points from the pixel plane into the real three-dimensional space [35,36], giving the image specific physical significance from which shaft-diameter information can be acquired. In this paper, to reduce errors caused by inaccurate calibration of the camera-based imaging system during the projection process, Zhang’s two-step calibration method [18] is adopted. This method ensures good calibration accuracy and requires a relatively simple calibration setup. In Zhang’s method, the equation shown as Equation (1) represents the mapping relationship between pixel coordinates in the image and the three-dimensional coordinates in the world coordinate system, as follows:

(1) $$Z_{C}\left[\begin{array}{c} u\\ v\\ 1 \end{array}\right]=\left[\begin{array}{cccc} \alpha & \gamma & u_{0} & 0\\ 0 & \beta & v_{0} & 0\\ 0 & 0 & 1 & 0 \end{array}\right]\left[\begin{array}{cc} R & t\\ 0^{T} & 1 \end{array}\right]\left[\begin{array}{c} X_{W}\\ Y_{W}\\ Z_{W}\\ 1 \end{array}\right]$$

where (*u*, *v*) represents the pixel coordinates of a two-dimensional point in the image, $\left(X_{W},Y_{W},Z_{W}\right)$ represents the three-dimensional world coordinates of the corresponding actual point, $Z_{C}$ represents the focal length, $A=\left[\begin{array}{ccc} \alpha & \gamma & u_{0}\\ 0 & \beta & v_{0}\\ 0 & 0 & 1 \end{array}\right]$ refers to the intrinsic matrix of the camera, $\left[\begin{array}{cc} R & t\\ 0^{T} & 1 \end{array}\right]$ refers to the extrinsic matrix of the camera, and t is the translation vector. $R=[r_{1}\;r_{2}\;r_{3}]$ is a rotation matrix composed of three mutually orthogonal column vectors.

The calibration of the imaging model is essentially the process of solving for the aforementioned parameters. In this section, Zhang’s two-step calibration method is utilized to calibrate the intrinsic and extrinsic parameters of the camera. This method involves calibrating the model by moving and rotating a one-dimensional planar target. The motion of the planar target is straightforward, requiring only manual movement and rotation of the calibration board to complete the calibration process. The algorithm consists of two main steps: first, obtaining initial parameter values using a linear model, and second, optimizing the initial parameters using a nonlinear model. Ultimately, the calibration process yields the intrinsic and extrinsic parameters of the camera, as well as distortion coefficients [37].

#### 2.1.2. Planar Calibration

**Figure 2:**
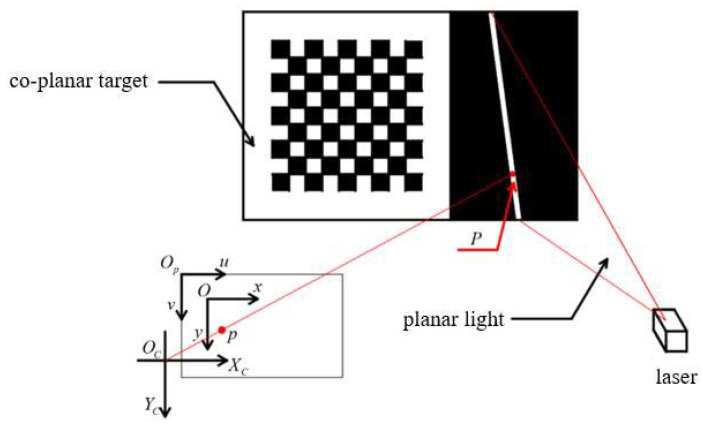
Schematic diagram of the principle underlying line-structured light plane calibration. Elements associated with structured light are shown in red.

The calibration of a planar line-structured light system involves solving for the equation that represents the central plane of the line-structured light. In a single-camera line-structured light-measurement system, only the two-dimensional information of the object being tested can be obtained from the CCD image. The depth information is constrained by the planar light; therefore, the accuracy of the calibration of the planar line-structured light directly affects the accuracy the obtained dimensional information of the object. The co-planar target-based planar calibration method has broad applicability and high calibration accuracy in the field of line-structured light vision. Therefore, this paper adopts the co-planar target-based method to calibrate the planar light. Figure 2 shows the chessboard co-planar target device used in this paper.

The fundamental principle of this method involves using the coplanarity property between the feature points of the line-structured light and the planar target to solve the equation of the planar light. As shown in Figure 2, the planar light beam emitted by the line laser forms a straight line on the chessboard co-planar target, and P represents an arbitrary center point on the bright line. Moving or rotating the co-planar target multiple times makes it possible to obtain a series of images of the co-planar target. The coordinates of the center points of the stripe in these images are located simultaneously in the target plane and the line-structured light plane in the camera coordinate system. Given the camera intrinsic parameters and distortion coefficients, the equation of the target plane in the camera coordinate system can be obtained using the coordinates of the corner points, and the camera coordinates of the center points of the bright lines can be obtained as well. Finally, using the camera coordinates of the center points of the bright lines from multiple images, the equation of the planar light in the camera coordinate system can be solved.

In the *j*-th co-planar target image, the camera coordinates of the *i*-th center point of the light stripe can be denoted as Ci,j = (Xi,j, Yi,j, Zi,j). Furthermore, it is assumed that the equation of the line-structured light plane in the camera coordinate system is given by

(2) $$b_{1}X_{C}+b_{2}Y_{C}+b_{3}Z_{C}+1=0$$

By substituting the camera coordinates of all center points of the light stripes into Equation (2) and expressing the system of linear equations in matrix form, we have:

(3) $$\left[\begin{array}{c} b_{1}\\ b_{2}\\ b_{3} \end{array}\right]={\left[\begin{array}{ccc} \sum_{j=1}^{N}\sum_{i=1}^{k}{\left(X_{C}^{j,i}\right)}^{2} & \sum_{j=1}^{N}\sum_{i=1}^{k}X_{C}^{j,i}Y_{C}^{j,i} & \sum_{j=1}^{N}\sum_{i=1}^{k}X_{C}^{j,i}Z_{C}^{j,i}\\ \sum_{j=1}^{N}\sum_{i=1}^{k}X_{C}^{j,i}Y_{C}^{j,i} & \sum_{j=1}^{N}\sum_{i=1}^{k}{\left(Y_{C}^{j,i}\right)}^{2} & \sum_{j=1}^{N}\sum_{i=1}^{k}Y_{C}^{j,i}Z_{C}^{j,i}\\ \sum_{j=1}^{N}\sum_{i=1}^{k}X_{C}^{j,i}Z_{C}^{j,i} & \sum_{j=1}^{N}\sum_{i=1}^{k}Y_{C}^{j,i}Z_{C}^{j,i} & \sum_{j=1}^{N}\sum_{i=1}^{k}{\left(Z_{C}^{j,i}\right)}^{2} \end{array}\right]}^{-1}\left[\begin{array}{c} \sum_{j=1}^{N}\sum_{i=1}^{k}X_{C}^{j,i}\\ \sum_{j=1}^{N}\sum_{i=1}^{k}Y_{C}^{j,i}\\ \sum_{j=1}^{N}\sum_{i=1}^{k}Z_{C}^{j,i} \end{array}\right]$$

By solving the aforementioned matrix equation, the parameters of the planar light equation can be obtained.

### 2.2. Image Enhancement

When conventional structured-light vision-measurement techniques are used to directly measure shaft components with highly reflective surfaces, the measurement results often exhibit significant deviations from the actual values. This deviation is due to the high surface reflectivity, which causes strong specular reflections on the part’s surface and results in the light being reflected in a specific direction. When the camera is positioned along the reflection path, the corresponding area in the image becomes overexposed, appearing to be pure white. Conversely, when the camera is not positioned along the reflection path, the corresponding area in the image becomes underexposed, appearing darker. Both situations lead to the loss of critical details in the image. Moreover, highly reflective objects are usually more sensitive to changes in the surrounding lighting conditions. Even small variations in ambient lighting can cause significant fluctuations in the grayscale values of the captured images. Sometimes, secondary reflections can form on the surface of the object and further affect the image quality. These secondary reflections disrupt the distribution of grayscale values in the projected light patterns, resulting in measurement results that deviate significantly from the true values.

**Figure 3:**
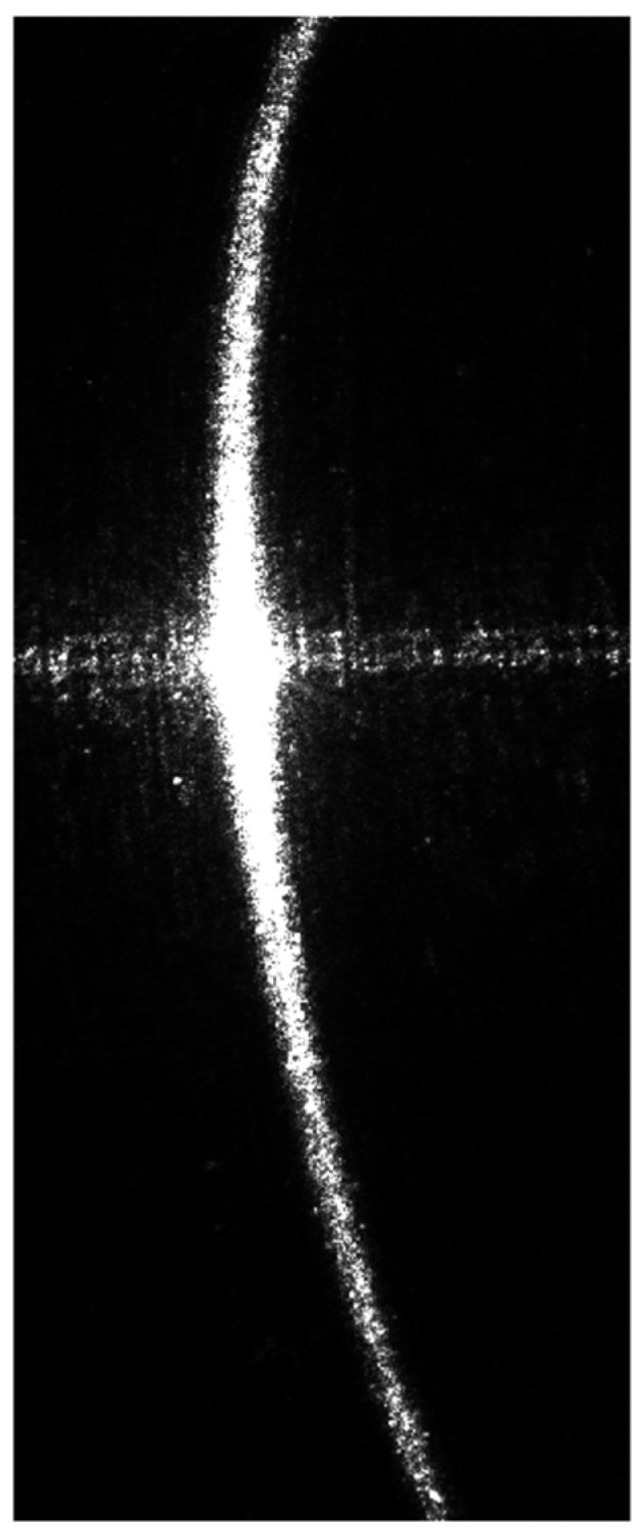
The structured light images of the surface of the section of a reflective steel shaft.

Figure 3 illustrates an example of a structured light image captured on a section of a reflective steel shaft. As shown, there are horizontal noise patterns in the middle of the image due to the strong surface reflectivity. The central part of the structured light is overexposed, while the light patterns on the sides are narrower and dimmer, exhibiting highly uneven quality. These issues pose significant challenges for measurement.

To overcome the challenges posed by surface reflections and achieve high-precision measurement of shaft components, this article proposes the following methods to enhance structured light images of reflective shafts and improve the quality of light patterns.

**Figure 4:**
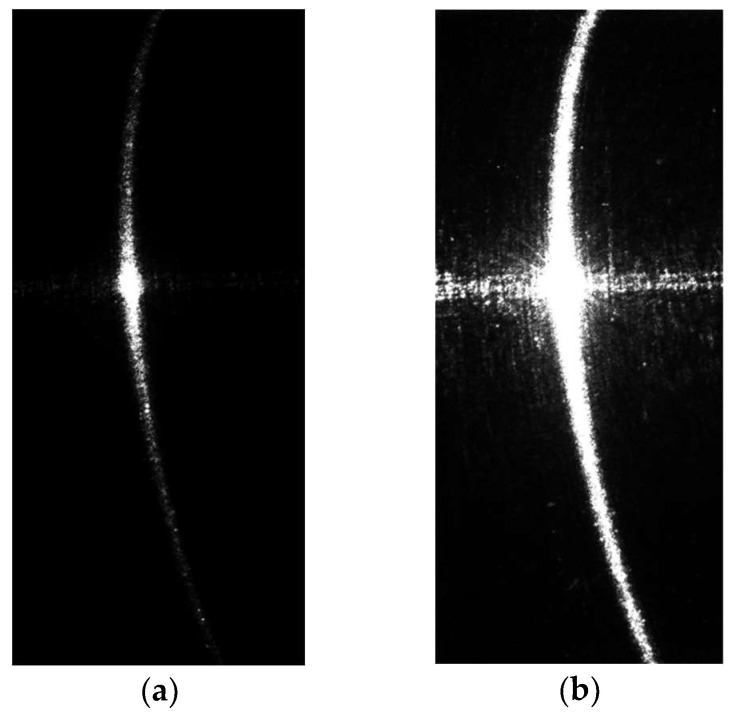
Schematic representation of light stripe images captured at different exposure times. (**a**) Light strip image taken at low exposure time. (**b**) Light strip image taken at high exposure time.

(1) Two structured light images with different exposure times were taken as shown in Figure 4. The lower exposure time ensures that there are no specular highlights or secondary reflections in the center of the image. The goal is to avoid overexposure and capture the details in the central region. On the other hand, higher exposure time ensures that the light patterns on both sides are bright and well-defined.

(2) Extract the center points of the light patterns from the image captured with the lower exposure time. If the center points at the ends of the light patterns are sparse, interpolation methods should be used to fill in the gaps and ensure continuity of the center points. Let the coordinate values of three adjacent points in the extracted center points be $\left(x_{i},y_{i}),(x_{i+1},y_{i+1}),(x_{i+2},y_{i+2}\right)$. If the condition $|x_{i}-x_{i+1}|>1$ is satisfied, then insert a new center point between them. This interpolation can be accomplished using the following formula:
  (4) $$\left\{\begin{array}{l} x=\frac{x_{i}+x_{i+1}}{2}\\ y=\frac{\left(x-x_{i})(x-x_{i+1}\right)}{\left(x_{i+2}-x_{i})(x_{i+2}-x_{i+1}\right)}y_{i+2}+\frac{\left(x-x_{i})(x-x_{i+2}\right)}{\left(x_{i+1}-x_{i})(x_{i+1}-x_{i+2}\right)}y_{i+1}+\frac{\left(x-x_{i+1})(x-x_{i+2}\right)}{\left(x_{i}-x_{i+1})(x_{i}-x_{i+2}\right)}y_{i} \end{array}\right\}$$

After inserting a new center point between two existing adjacent points, re-evaluate the distances between all adjacent points. Repeat this process of inserting new points and evaluating distances until the distance between any pair of adjacent points is less than 1.

(3) To assign weights to each pixel in the image, generate a weight map. This weight map assigns lower weights to points farther away from the center of the light pattern and greater weights to points closer to the center. The center points that were extracted and interpolated from the image captured with the low exposure time are denoted as $S_{i}(x_{i},y_{i}),\;i=1\cdots n$, and the coordinates of each pixel in the image are represented by $I_{j}=(x_{j},y_{j})$. The shortest distance $L_{j}$ between a pixel point and all the center points is calculated as follows:
  (5) $$L_{j}=\min\{\sqrt{{\left(x_{i}-x_{j}\right)}^{2}+{\left(y_{i}-y_{j}\right)}^{2}},i=1\cdots n\}$$

After calculating the shortest distance between a pixel and all center points, the weight value for pixel j can be computed using the following formula, where *σ* is a parameter adapted to the width of the light pattern:

(6) $$w_{j}=\frac{1}{\sqrt{2\pi }\sigma }\exp(-\frac{L_{j}^{2}}{2\sigma^{2}})$$

(4) To obtain the enhanced image, multiply the weight map by the high-exposure image. Let the grayscale value of pixel j in the high-exposure image be $G_{j}$. The adjusted grayscale value of pixel j in the enhanced image, denoted as $N_{j}$, can be calculated using the following formula:
  (7) $$N_{j}=G_{j}\cdot w_{j}$$

### 2.3. Shaft Diameter Measurement

The intersection between the structured light plane and the axial section forms an elliptical shape in three-dimensional space. This ellipse can be visualized as a planar ellipse in the structured light plane. The diameter of the measured shaft section corresponds to the length of the minor axis of this ellipse. Therefore, by converting the extracted pixel coordinates of the light-pattern center points into two-dimensional points on the plane of structured light, an ellipse fitting can be performed to determine the shaft diameter.

Let the general equation of the ellipse be:

(8) $$x^{2}+Axy+By^{2}+Cx+Dy+E=0$$

Let us denote the n two-dimensional points obtained after the coordinate transformation of the extracted center points from the captured light pattern image, projected onto the structured light plane, as $P_{i}(x_{i},y_{i})\;,i=1\cdots n$. According to the principle of least squares, the target function that needs to be fitted can be expressed as:

(9) $$F(A,B,C,D,E)=\sum_{i=1}^{n}{\left(x_{i}{}^{2}+x_{i}y_{i}A+y_{i}{}^{2}B+x_{i}C+y_{i}D+E\right)}^{2}$$

To minimize *F*, it is required that the partial derivatives of *F* with respect to the parameters are set to zero. This step leads to the following equations:

$$\frac{\partial F}{\partial A}=\frac{\partial F}{\partial B}=\frac{\partial F}{\partial C}=\frac{\partial F}{\partial D}=\frac{\partial F}{\partial E}=0$$

The regularized equation can be written as follows by substituting the coordinates of each point:

(10) $$\left[\begin{array}{ccccc} \sum x_{i}^{2}y_{i}^{2} & \sum x_{i}y_{i}^{3} & \sum x_{i}^{2}y_{i} & \sum x_{i}y_{i}^{2} & \sum x_{i}y_{i}\\ \sum x_{i}y_{i}^{3} & \sum y_{i}^{4} & \sum x_{i}y_{i}^{2} & \sum y_{i}^{3} & \sum y_{i}^{2}\\ \sum x_{i}^{2}y_{i} & \sum x_{i}y_{i}^{2} & \sum x_{i}^{2} & \sum x_{i}y_{i} & \sum x_{i}\\ \sum x_{i}y_{i}^{2} & \sum y_{i}^{3} & \sum x_{i}y_{i} & \sum y_{i}^{2} & \sum y_{i}\\ \sum x_{i}y_{i} & \sum y_{i}^{2} & \sum x_{i} & \sum y_{i} & N \end{array}\right]\left[\begin{array}{l} A\\ B\\ C\\ D\\ E \end{array}\right]=-\left[\begin{array}{l} \sum x_{i}^{3}y_{i}\\ \sum x_{i}^{2}y_{i}^{2}\\ {\sum x_{i}}^{3}\\ \sum x_{i}^{2}y_{i}\\ \sum x_{i}^{2} \end{array}\right]$$

For simplicity, the equation can be abbreviated as:

$$M_{1}\left[\begin{array}{l} A\\ B\\ C\\ D\\ E \end{array}\right]=M_{2}$$

The solution of the equation mentioned above will provide the five parameters of the ellipse equation:

$$\left[\begin{array}{l} A\\ B\\ C\\ D\\ E \end{array}\right]=M_{1}^{-1}M_{2}=M_{1}\backslash M_{2}$$

Once the above parameters are obtained, the geometric center of the ellipse can be calculated as follows:

$$X_{c}=\frac{AD-2BC}{4B-A^{2}}$$

$$Y_{c}=\frac{AC-2D}{4B-A^{2}}$$

Using this information, the measured shaft diameter, which corresponds to the semi-minor axis of the ellipse, can be calculated.

(11) $$b=2\sqrt{\frac{2(X_{c}^{2}+BY_{c}^{2}+AX_{c}Y_{c}-F)}{1+B-\sqrt{{\left(1-B\right)}^{2}+A^{2}}}}$$

**Figure 5:**
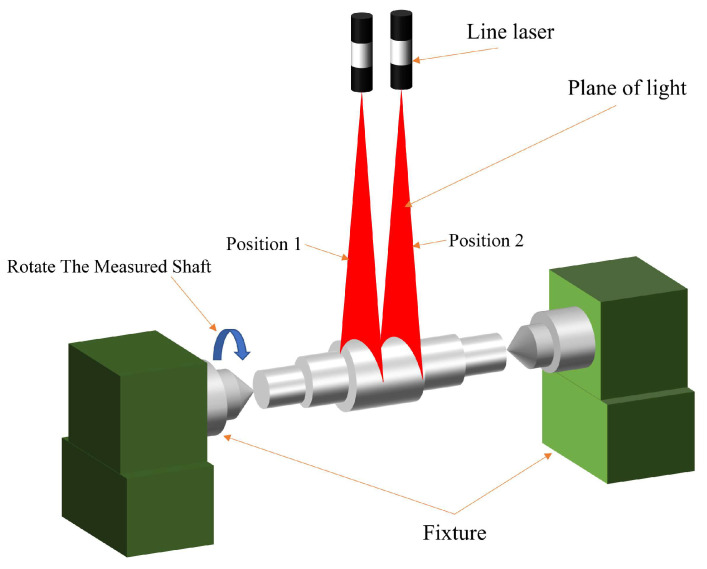
Improved model for measurement of shaft diameter.

In the process of machining shaft components, deviations from an ideal cylindrical shape may occur due to imprecision of the machining equipment, clamping errors, and cutting forces. On the cross-section where the light plane and the measured shaft intersect, the shapes on either side of the plane of symmetry may not match. On the overall shaft section, the diameter of each section will also be different due to the influence of cylindricity error. To address the impact of shape errors on the accuracy of diameter measurements, a shaft diameter measurement method is proposed, and the model of this method is shown in Figure 5. Due to the characteristics of the line-structured light-measurement model, the shape contour on one side of the measured shaft can be obtained using a single-line laser. First, the measured shaft is fixed through the double apex. Next the shaft rotates around the line connecting the double apex. Rotating the shaft allows multiple light stripe images to be obtained on the same section of a measured shaft. The process ensures that these light stripe images can cover the contour information on the cross-section of the measured shaft. The diameter of the measured shaft corresponding to each light strip is calculated, and the average of these diameters is taken as the diameter of the shaft in that section. When using the machining and measurement system for shaft parts, it is necessary to ensure that the light plane is as perpendicular as possible to the axis of the measured shaft during measurement. Secondly, in order to reduce the impact of cylindricity error on the measurement of shaft diameter, the line laser is moved multiple times. As shown in Figure 5, the line laser is moved from position 1 to position 2, and the shaft diameter corresponding to position 2 is obtained by repeating the measurement steps at position 1. Finally, the overall information of the measured shaft segment is obtained by moving the line laser multiple times, and the average diameter of all cross-sections is taken as the diameter of the shaft segment.

## 3. Experiments and Results

**Figure 6:**
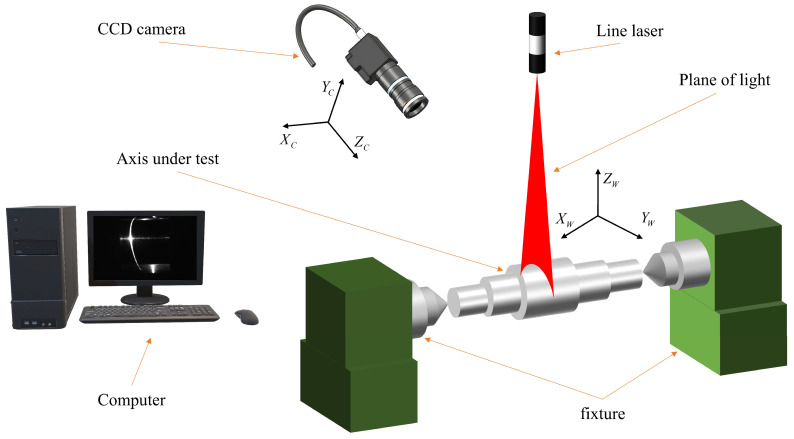
Schematic of the experimental site.

In order to fully demonstrate the effectiveness of the proposed method, the methods and procedures described above will be applied in this study to measure the diameter of a reflective steel shaft section using linear structured light. The acquired reflective images will be processed, and the measurement results obtained before and after processing will be compared. The experimental setup used at the test site is shown in Figure 6. The specific parameters of the experimental apparatus employed in this study are presented in Table 1 below.

### 3.1. Camera Calibration

**Figure 7:**
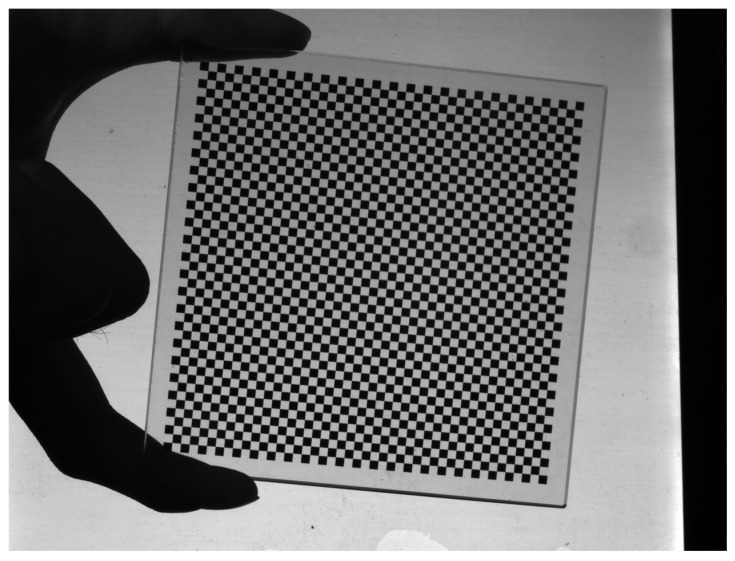
Calibration image with checkerboard calibration board.

The calibration of the industrial camera was performed using Zhang’s two-step calibration method. The calibration board used in this study was a chessboard pattern, as shown in Figure 7

Camera calibration was conducted using nine chessboard-pattern images captured at different orientations. The calibration results are as Table 2:

In the table, the variables α, β, γ, $u_{0}$, $v_{0}$ represent the five intrinsic parameters of the camera. The parameters k1, k2, p1, and p2 correspond to the four coefficients for radial and tangential distortion. The coefficient *re* denotes the reprojection error of the camera calibration, which is measured in pixels.

### 3.2. The Calibration of the Light Plane

**Figure 8:**
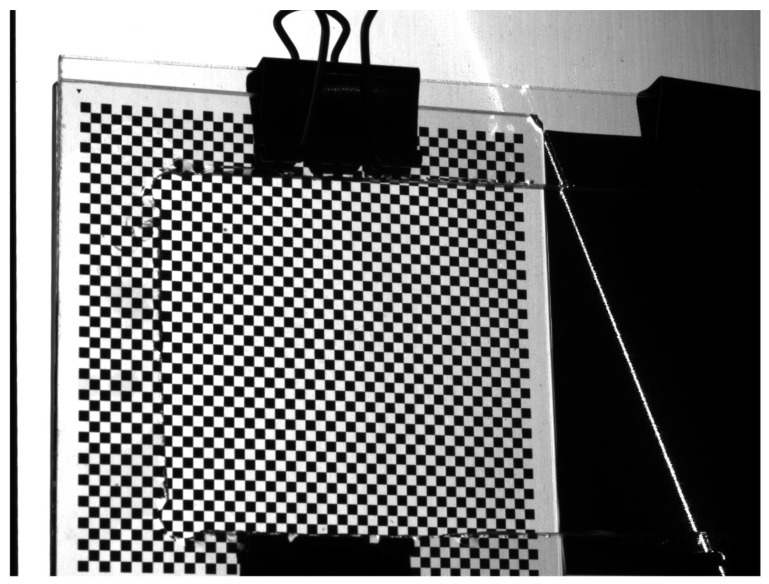
Coplanar calibration target for optical plane calibration.

In this study, a coplanar target was used to calibrate the structured light plane, as shown in Figure 8. Calibrating the target allowed the camera coordinates of the structured light on the target to be obtained. Fitting multiple structured light stripes with known camera coordinates allowed the equation of the structured light plane to be determined.

The calibration of the light plane was completed using six images of the coplanar target captured at different orientations. The equation representing the calibrated light plane is as follows:

(12) 2.4656x+0.5433y+1.4717z−1000=0

### 3.3. The Enhancement of the Structured Light Stripe Images

**Figure 9:**
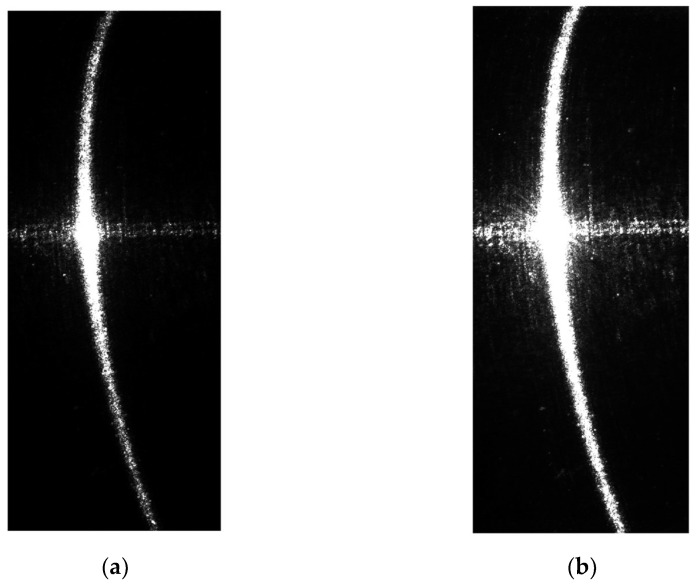
Structured light stripe images with different exposure times: (**a**) is the low-exposure image, with an exposure time of 30 ms; (**b**) is the high-exposure image, with an exposure time of 300 ms.

As in the above-described procedure, the first step is to capture two structured light stripe images with different exposure times, as shown in Figure 9.

**Figure 10:**
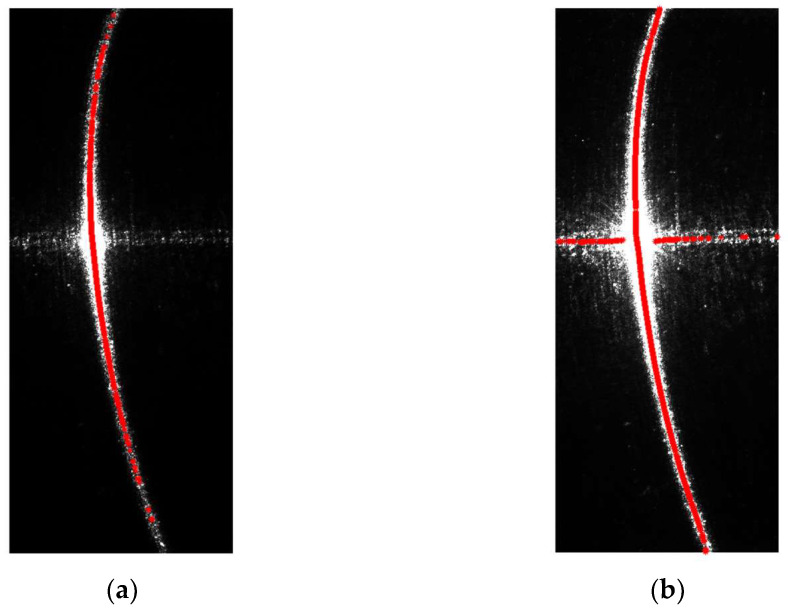
Results of the extraction of the center points of the structured light stripes in the original images: (**a**) represents the center points of the structured light stripes in the low-exposure image; (**b**) represents the center points of the structured light stripes in the high-exposure image.

If the center points of the structured light stripes are directly extracted from the original images, they will contain a significant amount of noise, which would lead to unreliable measurement results. The center points extracted from the original light stripes are shown in Figure 10. It can be observed that in the low-exposure image, although the influence of surface specular reflections is relatively small and there is no lateral noise in the middle, the center points of the light stripes on the two sides are not continuous due to the presence of darker regions at the ends. This discontinuity results in a loss of detail on the sides. Conversely, in the high-exposure image, severe lateral noise occurs in the middle, while the light stripes on the sides remain continuous.

**Figure 11:**
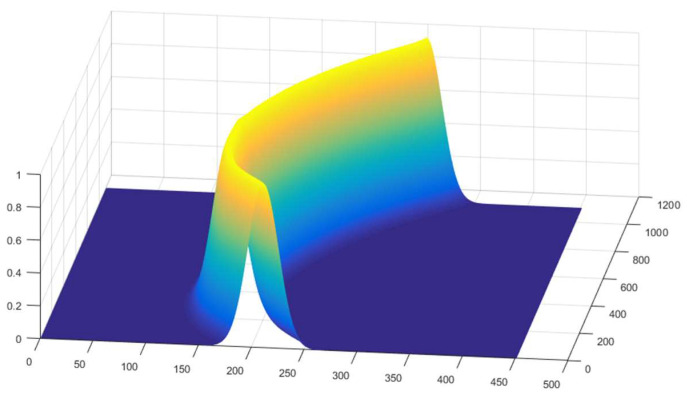
Weight maps of the structured light stripe images. Colors represent weights, and warmer colors represent higher weight values.

Following the method described above, weight values were calculated for each pixel in the image. The weights were based on the center points of the light stripes in the low-exposure image, which contained less noise. The resulting weight map, which consists of weight values for all pixels, is shown in Figure 11.

**Figure 12:**
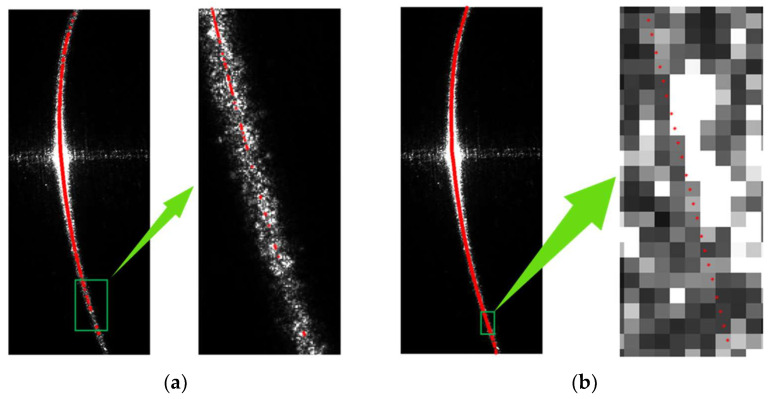
Comparison of the images showing the distribution of center points before and after interpolation. (**a**) is the distribution of center points in the image with an exposure time of 30 ms; (**b**) is the distribution of center points in the processed image.

**Figure 13:**
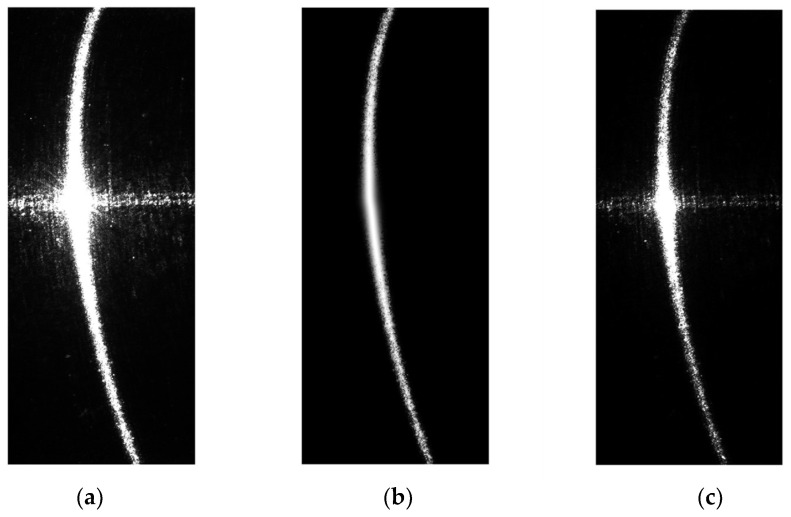
Comparison between the enhanced light stripe image and the pre-enhanced image. (**a**) is the image of the structured light stripe in high-exposure image; (**b**) is the processed image of the structured light stripe; (**c**) is the image of the structured light stripe in the low-exposure image.

Multiplying the weight map by the high-exposure light stripe image results in the final image, which is shown in Figure 12 and Figure 13. Figure 12 shows the comparison of the distribution of light-stripe center points before and after interpolation, and Figure 13 shows the comparison of light stripe images before and after processing.

**Figure 14:**
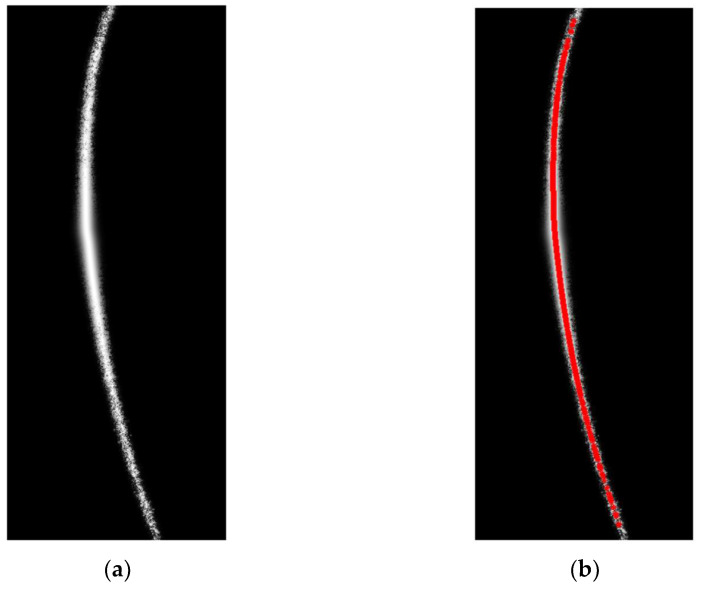
Final enhanced image and results of center-point detection for the structured light stripes: (**a**) is the final enhanced image; (**b**) is the results of center-point detection for the structured light stripes in the final image.

An image representing the light-stripe center points in the final image is shown in Figure 14b. It is evident that the light stripe image processed using the method described in this paper combines the advantages of the low-exposure and high-exposure light stripe images. The overall image quality is uniform, surface reflections are suppressed, the extracted light-stripe center points are more continuous, and the noise is significantly reduced.

### 3.4. The Measurement Results of the Shaft Diameter Ignoring Shape Errors

To validate the improvement in measurement accuracy of the proposed method for measuring the diameter of a reflective shaft, multiple measurements of the diameter of the section of the reflective shaft were conducted using the aforementioned method. The measurement results were compared with the measurements obtained from the original, unenhanced images. Furthermore, all measurement values were compared against the ground truth of the diameter to validate the accuracy of the measurements. The ground truth was obtained through a least-squares cylindrical fitting of the full data acquired from a three-coordinate measuring instrument (CMM).

The diameter of the same reflective shaft section was measured at different orientations, with a total of six measurements conducted. For each measurement, two structured light images with different exposure times were captured and used for diameter measurement using the low-exposure image, the high-exposure image, and the enhanced image obtained through the proposed method. The obtained measurement results were as shown in Table 3.

Table 3 shows the results of the multiple measurement experiments conducted on a section of a reflective steel shaft. The light stripe images captured with a low exposure time are less affected by reflections, with an average measurement error of 60 μm. However, due to the lack of detailed information at the ends of the light stripes, the measurement accuracy is generally lower. On the other hand, the light stripe images captured with high reflectivity include high intensity at the ends but are significantly affected by surface reflections in the middle, leading to significant noise and highly inaccurate measurement results. These images do not provide reliable measurements. The image quality was significantly enhanced using the method proposed in the paper. The processed images combine the advantages of the high- and low-exposure-time images, complementing each other’s shortcomings. As a result, the measurement accuracy is significantly improved, with an average error of only 11 μm. The improvement results in an accuracy approximately five times that of the low-exposure image, while the measurement time is only doubled. This result confirms the effectiveness of the method proposed in this paper.

In order to verify the measurement precision of the method proposed in the paper, the root mean squared error (RMSE) of six measurements was calculated. Compared to the low-exposure and high-exposure images before processing, the measurement results corresponding to the enhanced image have the smallest root mean square error, at 10.98 μm. The measurement results demonstrate that the method proposed in the paper improves the accuracy and precision of shaft-diameter measurement based on line-structured light vision.

### 3.5. The Measurement Results of the Shaft Diameter with Shape Errors

Due to the inherent characteristics of the line-structured light-measurement system, the elliptical fitting measurement of the shaft diameter utilized only a portion of the contour of the measured shaft surface. To mitigate the influence of machining errors on the estimated diameter caused by non-ideal shapes, additional experiments were conducted to obtain more realistic estimates of the shaft diameter. For the diameter measurement of a single shaft segment, different light planes were calibrated to obtain estimates of the shaft diameter for three different cross-sections of the shaft. Furthermore, the measured shaft was clamped on a dual-tip measuring table and rotated at three different angles to acquire the contours of different regions of the same cross-section, along with corresponding estimates of the shaft diameter. The average of the diameter estimates obtained at different positions and angles was taken to obtain a more realistic final measurement value.

The results of camera calibration for the supplementary experiments are presented in the Table 4.

The calibration results for the three different light planes for the cross-sections are as follows:

(13) Light plane 1:       3.2831x+0.0989y+1.5003z−1000=0

(14) Light plane 2:       3.1749x+0.1384y+1.5231z−1000=0

(15) Light plane 3:       3.2748x+0.0692y+1.6048z−1000=0

**Figure 15:**
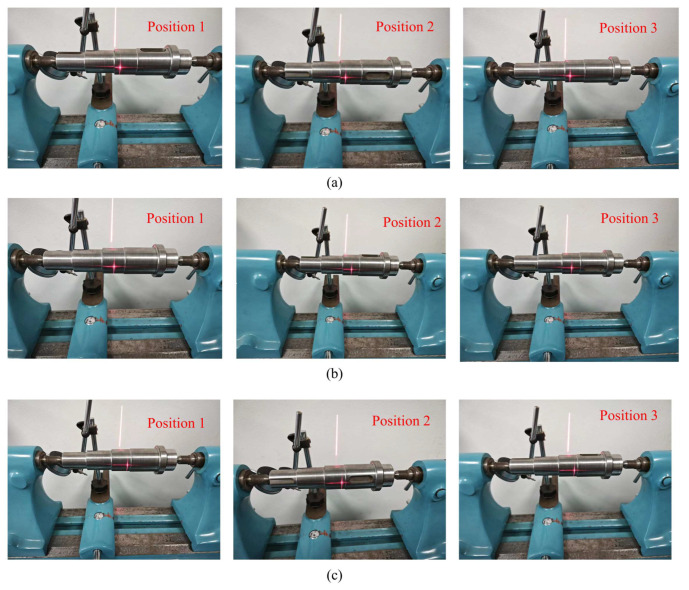
Surface images of the measured shaft (shaft with high surface accuracy). (**a**) The left section. (**b**) The middle section. (**c**) The right section.

**Figure 16:**
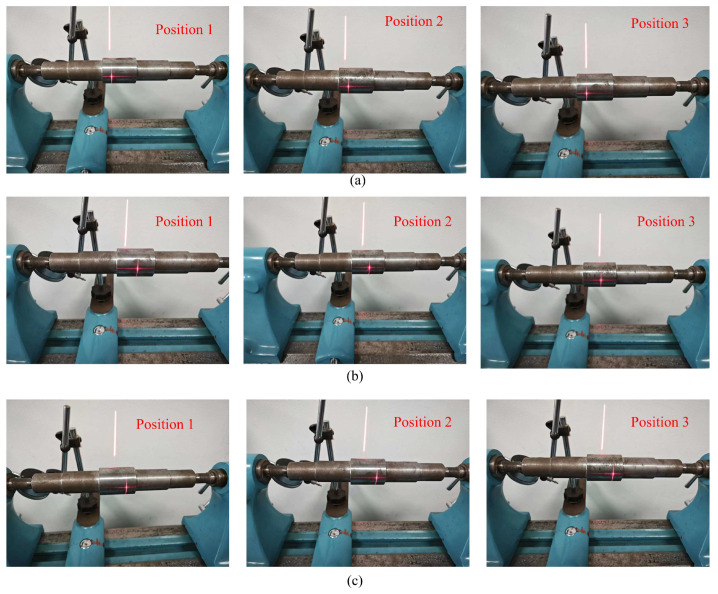
Surface images of the measured shaft (shaft with low surface accuracy). (**a**) The left section. (**b**) The middle section. (**c**) The right section.

The estimated diameter values obtained at different positions and angles, along with the corresponding measurements, are listed in the table below. The same measurement process was applied to two shafts with different surface qualities and diameters. To provide a more intuitive evaluation of the measurement results, the absolute errors of the measurements are listed in Table 5 and Table 6. The images of light stripes corresponding to different cross-sections of each shaft segment are shown in Figure 15 and Figure 16.

From Table 5 and Table 6, it can be observed that due to the less-than-ideal surface shape of the components, there are objective differences in various regions, with variations in the estimated axial diameters at different positions and angles. Variations are especially notable near the true values of the axial diameter. It is necessary to consider estimates from different positions and angles and provide a final measurement value by synthesizing these values. We present the mean estimates of axial diameters at different angles and cross-sections within the same axial segment as more compelling final measurements of axial diameter. For both pre-enhancement low-exposure images and pre-enhancement high-exposure images, considering the means of measurements taken at various positions indeed brings the measurement results closer to the true values; however, significant errors persist, especially in the case of high-exposure images. In contrast, our proposed method provides enhanced images with consistently superior accuracy, demonstrating the effectiveness and robustness of the approach.

## 4. Conclusions

In response to the problem of inaccurate measurement of key dimensions of reflective objects that arises in current structured light vision measurement techniques due to the influence of surface reflections, this paper proposes a method based on light stripe image restoration and enhancement. The method aims to improve the quality of structured light stripe images captured by industrial cameras, suppress the impact of surface reflections on image quality, and enhance measurement accuracy. The measurement system is calibrated using a chessboard calibration board and a coplanar target, establishing the mapping relationship between the two-dimensional pixel points in the image and the true three-dimensional points on the object surface. Extracting the light-stripe centers from the low-exposure image allows pixel-wise weight values to be calculated to create a weight map. This weight map is used to perform grayscale correction on the high-exposure image, pixel by pixel. This process preserves the quality of the light stripes while suppressing the effects of surface reflections. As a result, it improves the accuracy of detection of the light stripe center and significantly enhances the accuracy of measurement of shaft diameters. In the measurement experiments, the proposed method achieved measurement results that, on average, differed by only 11 μm from those obtained by CMM. The maximum difference was 18 μm, while the minimum difference was only 7 μm. The measurement accuracy of the proposed method was six times higher than that of the unprocessed light stripe images. The utilization of the average of measurements from different positions and angles to estimate the shaft diameter further improved the precision and robustness of the measurements.

## Figures and Tables

**Table 1:** Experimental equipment and parameters.

CCD camera	Model: MER-125-30UM				
	photosensitive unit	frame rate	resolution	size	image sensor
	3.75 × 3.75 μm	30 f/s	1292 × 964 pixel	29 × 29 × 29 mm	1/3″grayscale
Optical lens	Model: Computar M2514-MP				
	aperture	interface	focal length	aperture ratio	working temperature
	F1.4-F16C	C-type	25 mm	1:1.4	−20–50 °C
Calibration board	Model: NANO CBC 75 mm-2.0				
	shape	graphic accuracy	accuracy	Shape size	grid size
	checkerboard	±1.0 μm	Level 1	75 × 75 × 3.0 mm	2.0 × 2.0 mm
Laser	Model: LH650-80-3				
	size	color	wavelength	power	exit pupil diameter
	ф 16 × 45 mm	red light	650 nm	0~20 mW	Ф 8 mm
Backlight	Model: CCS LFL-200				
	color	installation size	power	external dimensions	light-emitting area
	red	200 × 212 mm	12 V/10 W	234 × 222 mm	200 × 180 mm

**Table 2:** Camera-Calibration Results.

Camera Intrinsic Parameters		Distortion Coefficients	
α	18,607.65	k1	−0.0085
β	18,690.94	k2	1.5291
γ	−42.20	p1	0.0003
$u_{0}$	3413.44	p2	0.0004
$v_{0}$	1587.62	*re*	0.005

**Table 3:** Results of measurement of the diameter of the reflective shaft segment. (Unit: mm).

	1	2	3	4	5	6	Mean Value	Root Mean Squared Error
Low-exposure image	39.801	39.780	39.922	39.896	39.792	39.806	39.8330	0.05497
absolute error	0.071	0.083	0.068	0.032	0.053	0.055	0.06	
High-exposure image	41.107	40.602	43.156	42.106	40.997	43.264	41.872	1.04920
absolute error	1.253	1.748	3.302	2.252	1.143	3.41	2.185	
Processed image	39.872	39.863	39.841	39.864	39.845	39.861	39.858	0.01098
absolute error	0.018	0.009	0.013	0.010	0.009	0.007	0.011	
ground truth	39.854							

**Table 4:** Results of camera calibration for the supplementary experiment.

Camera Intrinsic Parameters		Distortion Coefficients	
α	13,622.09	k1	−0.0651
β	13,604.25	k2	1.1355
γ	21.13	p1	0.0011
$u_{0}$	1879.12	p2	0.0011
$v_{0}$	1417.97	*re*	0.0576

**Table 5:** Results of the shaft-diameter measurement from the supplementary experiment (unit: mm).

		Position1				Position2				Position3				Mean	RMSE
		Angle 1	Angle 2	Angle 3	Mean	Angle 1	Angle 2	Angle 3	Mean	Angle 1	Angle 2	Angle 3	Mean		
shaft1	Low-exposure	40.031	40.062	40.043	40.0453	40.122	40.125	40.119	40.1220	40.113	40.196	40.098	40.1357	40.1010	0.0475
	High-exposure	41.045	41.067	41.030	41.0473	40.094	41.255	41.452	40.9337	42.003	41.762	41.411	41.7253	41.2354	0.5115
	Processed image	40.064	40.072	40.059	40.0650	40.088	40.103	40.095	40.0953	40.092	40.096	40.089	40.0923	40.0842	0.0145
	Ground truth	40.080													
shaft2	Low-exposure	39.934	40.024	40.009	39.9890	40.020	40.017	39.982	40.0063	40.016	40.012	40.007	40.0117	40.0023	0.0267
	High-exposure	41.438	42.054	42.047	41.8463	40.099	41.768	41.574	41.1470	42.073	41.067	42.068	41.7360	41.5764	0.6179
	Processed image	40.031	40.039	40.043	40.0377	40.058	40.066	40.059	40.0610	40.073	40.059	40.077	40.0697	40.0561	0.0147
	Ground truth	40.050													

**Table 6:** Absolute errors of shaft-diameter measurements from the supplementary experiment (unit: mm).

		Position 1				Position 2				Position 3				Mean	RMSE
		Angle 1	Angle 2	Angle 3	Mean	Angle 1	Angle 2	Angle 3	Mean	Angle 1	Angle 2	Angle 3	Mean		
shaft1	Low-exposure	0.0490	0.0180	0.0370	0.0347	0.0420	0.0450	0.0390	0.0420	0.0330	0.1160	0.0180	0.0557	0.0441	0.0274
	High-exposure	0.9650	0.9870	0.9500	0.9673	0.0140	1.1750	1.3720	0.8537	1.9230	1.6820	1.3310	1.6453	1.1554	0.5115
	Processed image	0.0160	0.0080	0.0210	0.0150	0.0080	0.0230	0.0150	0.0153	0.0120	0.0160	0.0090	0.0123	0.0142	0.0052
shaft2	Low-exposure	0.1160	0.0260	0.0410	0.0610	0.0300	0.0330	0.0680	0.0437	0.0340	0.0380	0.0430	0.0383	0.0477	0.0267
	High-exposure	1.3880	2.0040	1.9970	1.7963	0.0490	1.7180	1.5240	1.0970	2.0230	1.0170	2.0180	1.6860	1.5264	0.6179
	Processed image	0.0190	0.0110	0.0070	0.0123	0.0080	0.0160	0.0090	0.0110	0.0230	0.0090	0.0270	0.0197	0.0143	0.0068

## Data Availability

Data are contained within the article.
